# Research on a Trustworthiness Measurement Method of Cloud Service Construction Processes Based on Information Entropy

**DOI:** 10.3390/e21050462

**Published:** 2019-05-02

**Authors:** Gao Tilei, Li Tong, Yang Ming, Jiang Rong

**Affiliations:** 1 School of Information, Yunnan University of Finance and Economics, Kunming 650221, China; 2 Key Laboratory in Software Engineering of Yunnan Province, Kunming 650091, China; 3 Big Data School, Yunnan Agricutural University, Kunming 650201, China

**Keywords:** cloud service constructing process (CSCP), trustworthiness measurement, information entropy

## Abstract

The popularity of cloud computing has made cloud services gradually become the leading computing model nowadays. The trustworthiness of cloud services depends mainly on construction processes. The trustworthiness measurement of cloud service construction processes (CSCPs) is crucial for cloud service developers. It can help to find out the causes of failures and to improve the development process, thereby ensuring the quality of cloud service. Herein, firstly, a trustworthiness hierarchy model of CSCP was proposed, and the influential factors of the processes were identified following the international standard ISO/IEC 12207 of the software development process.Further, a method was developed combined with the theory of information entropy and the concept of trustworthiness. It aimed to calculate the risk uncertainty and risk loss expectation affecting trustworthiness. Also, the trustworthiness of cloud service and its main construction processes were calculated. Finally, the feasibility of the measurement method were verified through a case study, and through comparing with AHP and CMM/CMMI methods, the advantages of this method were embodied.

## 1. Introduction

With the development and rapid popularization of cloud computing, cloud service has become widely accepted as a new computing model. Cloud service refers to a type of emerging network service relying on a cloud computing platform. Its outsourcing service model and the security risk of the cloud platform itself have aroused users’ concerns about its trustworthiness. Therefore, how to build secure and trustworthy cloud services has become one of the hotspots in the research field over the past years [1]. The field of software process research believes that process plays a determinant role in product quality [2]. Therefore, to improve the trustworthiness of cloud services, the trustworthiness problems in the process must be solved. 

There are mainly two research directions on process trustworthiness: process measurement and process improvement methods. Reference [3] considered software process assessment (SPA) as a foundation step for software process improvement. For improving and optimizing the software process, the first step is to measure the software process objectively and find out the problematic process. Otherwise, insufficient process improvement and optimization may cause the failure of the process [4]. Main research results in process measurement are CMM [5] and CMMI [6] models developed by the United States Department of Defense, the Software Engineering Institute (SEISM) of Carnegie-Mellon University and the National Defense Industry Association. The two models classified the development stages of software organizations in practice as defining, implementing, measuring, controlling and improving software products. However, only a framework was put forward, extracting no specific knowledge of each key process area and failing to quantify the quality of a specific process. Also, the two models are primarily used to evaluate the degree of process management practices of a development organization, involving many contents, and being time-consuming and costly. As a result, small and medium-sized software companies, even some large ones, face challenges to meet relevant requirements and standards. For most software development organizations, what they need is an objective, quantitative, real and easy-to-implement measurement method. The method should enable them to find the weak links in the cloud service construction process (CSCP), and then carry out subsequent improvement or reinforcement, thus reducing the probability of institutional failure and improving product quality. Other primary software process measurement methods include Goal-driven Software Measurement (GSM) [7], Practical software measurement (PSM) [8,9], and Statistical Process Control (SPC) [10]. Based on the characteristics of the CMM and GQM model [11], reference [12] established a software process framework supporting metrics and gave the metrics of software process improvement. However, it did not give the exact measurement steps for the software development process. Reference [13] discussed the significant problems in software process measurement and presented an active measurement model (AMM) to support software process improvement (SPI). It emphasized the measurement of quality, maintainability and stability of the software products, rather than the processes. To find the problems in the product, we still need to reverse the development or maintenance processes for finding the processes responsible for the problems. 

To this end, a trustworthiness measurement method for CSCP based on information entropy is proposed here. Through in-depth study of CSCP, the risk factors in CSCP are analyzed and summarized. Then, according to the frequency of each risk factor and the degree of corresponding loss, the uncertainty of the primary construction process for cloud services and the expectation of risk loss are calculated by using information entropy. Finally, process trustworthiness is calculated based on the relevant concepts. Development, maintenance, and other processes are directly measured. By comparing the uncertainty and loss expectations of each process and the whole process, the organization can find the weaknesses and influential factors in CSCP. The present study provides an excellent method to help organizations reduce risk, optimize and improve the development and construction process, thereby fundamentally enhancing the trustworthiness of cloud service products. The paper is structured as follows:

Section 1: Introduction
Section 2: Related work
Section 3: Framework of CSCP
Section 4: CSCP Trustworthiness Measurement Method
Section 5: Case Study and Analysis
Section 6: Conclusions.

## 2. Related Work

### 2.1. Cloud Computing and Cloud Services

Cloud computing has become one of the research hotspots in the current computer field [14], and a model for enabling ubiquitous, convenient, on-demand network access to a shared pool of configurable computing resources [15]. To deeply study cloud computing and reduce its complexity, NIST divided cloud computing into three levels according to service types: Software as a Service (SaaS), Platform as a Service (PaaS), and Infrastructure as a Service (IaaS) [15]. On this basis, Linthicum further proposed the concept of “service stack”, and summarized 11 service modes of cloud computing, including storage as a service, database as a service, process as a service, information as a service, application as a service, platform as a service, integration as a service, security as a service, management as a service, test as a service, infrastructure as a service [16]. In a sense, cloud computing is cloud services or service computing. For the future of cloud services or service computing, Chen and Zheng [17] believed that there were two main development directions in the future: one was to build a large-scale underlying infrastructure closely integrated with applications, so that applications could expand to a large scale; the other was to build new cloud computing applications to provide a richer user experience on the network.

In the aspect of the analysis and design of the system model of SaaS services, Meng at Jinan University has put forward a seven-layer model based on the traditional software development five-level model to meet the needs of SaaS software development [18]. It was the early study of SaaS service at the level of the development model; Yuan in his paper “Research of online software system development solution based on SaaS” [19] had studied the modeling methods, security handling and database design of SaaS software. At the database level, many studies have been done wherein the data storage method of SaaS software and the way of expanding the data storage were the main direction, such as Zha [20] and Yu [21], both of whom have proposed a solution to the data structure of SaaS service. 

Cloud-native applications is a good cloud computing design pattern, which has offered a unique blend of academic knowledge and practical experience due to a variety of authors [22]. It is common sense that cloud-native applications (CNA) are intentionally designed for the cloud [23]. Reference [24] has described the key technologies for cloud native design to meet the requirements of a successful cloud application, including dynamic scalability, extreme fault tolerance, seamless upgradeability and maintenance and security are the basic properties of successful cloud applications. Reference [25] has presented a reference model for cloud-native applications that relies only on a small subset of well standardized IaaS services, which can be used to guide technology identification, classification, adoption, research and development processes for cloud native application and for vendor lock-in aware enterprise architecture engineering methodologies.

The existing research on cloud computing and cloud services is mostly focused on cloud services themselves and the research about the quality of cloud services mainly starts from the environment, content, development method, structure and design mode, ignoring the specific development environment and development process of cloud services. Osterweil [26] has pointed out that software processes are also software too, and that software process is of the same importance as software itself. Later, software process technology has proved to be effective in the support of many business activities [27]. 

For the same method and structure, different development organizations and different development processes may produce products of different quality. Therefore, besides paying attention to the development method and structure of service itself, to improve the quality of a specific service, measuring and optimizing its development and construction process is a very important link. 

### 2.2. Trustworthy Cloud Service

In terms of the factors affecting trustworthiness, [28] summarized the non-functional requirements of the reliable software proposed by relevant scholars and institutions since the 1985 as trusted computer system evaluation criteria (TCSEC). It was concluded that there was still not any recognized standard for the non-functional requirements of reliable software. Thus, a non-functional requirements decomposition model for reliable software was proposed, and concerns and soft goals were obtained, based on the Software Trustworthiness Classification Specification (STC 1.0) and the software quality requirements defined by ISO/IEC 25010 [29]. Reference [30] presented a model targeting both quantitative and qualitative non-functional properties (NFPs) and developed two algorithms to obtain the NFPs. The research findings mentioned above laid a solid foundation for the present study when analyzing the factors influencing the trustworthiness of CSCP. Reference [31] conducted an in-depth investigation of the trustworthiness and risk in Web services, identifying the relationship between trustworthiness and risk. Through the analysis and study of process risk factors, the calculation method of process trustworthiness was obtained in this paper.

Regarding service measurement and selection, [32] demonstrated the concerns of potential users on the trustworthiness of cloud computing service, hindering the development of cloud computing. Thus, a trustworthy cloud service attribute model was developed, and a trustworthiness evaluation method was put forward based on Information Entropy and Markov Chain. Reference [33] also applied information entropy and Markov Chain method into the calculation of the risks in cloud services. It also highlighted the high impact from the uncertainty of risks on cloud computing. Reference [34] proposed an information-entropy-based decision-making method for selecting cloud computing service. To help select trustworthy services, [35] developed a support vector machine (SVM)-based collaborative filtering (CF) service recommendation approach. In [36], starting from requirements for users’ privacy protection, a cloud service evaluation model based on trustworthiness and privacy-awareness was put forward. Combining objective and subjective trustworthiness assessment, [37] designed an integrated trustworthiness evaluation method.

Other areas of research in trustworthy computing include trustworthy computing models in IoT [38], trustworthy computing problems in SOA [39], the influence of uncertainty on trustworthy computing [40], trustworthy computing problem in the social network [41,42,43,44], and so on.

The existing research fields of trustworthy cloud computing covered a wide range, but with some distinct emphases. Some studied the factors affecting the trustworthiness including uncertainty; some explored the methods of measuring and selecting the trustworthy cloud services from the perspective of risk. Few studies of trustworthiness focused on the CSCP determining the trustworthiness of cloud services. Therefore, based on previous trustworthiness researches, herein the processes of constructing cloud services were analyzed to establish a trustworthiness measurement hierarchy model for cloud service construction process. Furthermore, the trustworthiness of the construction process was measured based on the theory of information entropy.

### 2.3. Information Entropy

Shannon introduced the concept of physical entropy into information theory and defined the magnitude of information, named information entropy. Information entropy can be used to measure the amount of message information in communications effectively. The greater the entropy, the higher the uncertainty and the less the amount of information. Despite various understandings of risk, the uncertainty of risk is commonly accepted, which is the essence of risk. The greater the uncertainty, the greater the risk. This essential feature of risk is consistent with the concept of information entropy. Thus, information entropy was selected here to measure the risk and trustworthiness of CSCP. To put it simply, the greater the uncertainty, the greater the entropy, the greater the risk, and thus the lower the trustworthiness. The definition of entropy [45] is given below:

**Definition 1** **(Information entropy)** *Let X be a discrete random variable, and n is the number of its possible values, then X = {x_1_, x_2_… x_n_}. For each x_i_, its probability value is P(x_i_), and the discrete probability space is:*

$$\left[\begin{array}{c} X\\ P\left(x\right) \end{array}\right]=\;\left[\begin{array}{ccc} x_{1} & \ldots & xn\\ P\left(x_{1}\right) & \ldots & P\left(x_{n}\right) \end{array}\right]$$

*Then:*

(1) $$H\left(X\right)=\;-\;\overset{n}{\underset{i=1}{\sum }}P\left(x_{i}\right)logP\left(x_{i}\right)$$

*H (X) is called information entropy of discrete random variable X.*

Information entropy is used to describe the uncertainty of things. For CSCP, the frequency of impact on the trustworthiness of the service is uncertain. The higher the uncertainty degree of different influencing factors is, the higher the risk will be, and the lower the trustworthiness will be. It is in line with the concept and characteristics of information entropy, which hence was chosen as a measure of uncertainty in CSCP.

## 3. Framework of CSCP

According to ISO/IEC 12207 [46] information technology and its improved application [47], the software life cycle process is divided into three categories: main process, supporting process and organizing process. Despite the service nature, cloud services are a new form of software in the new computing era.

**Figure 1:**
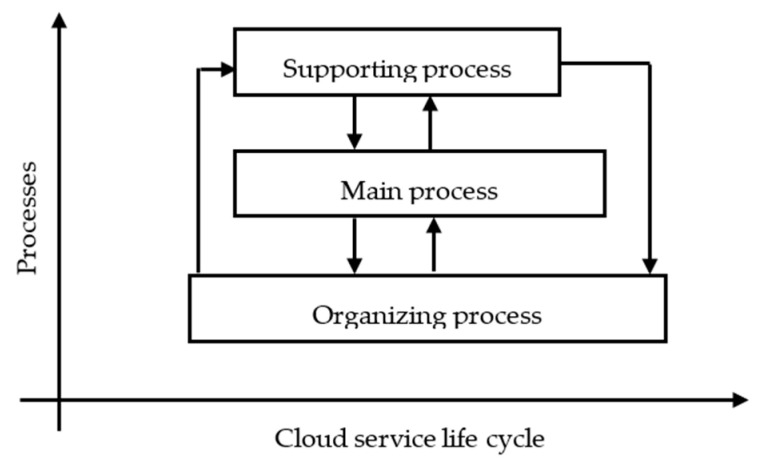
Processes in CSCP.

**Figure 2:**
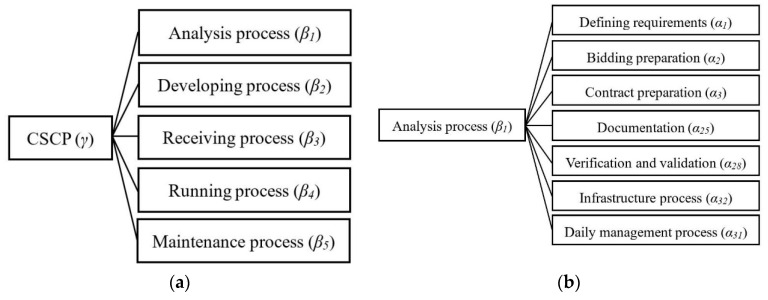

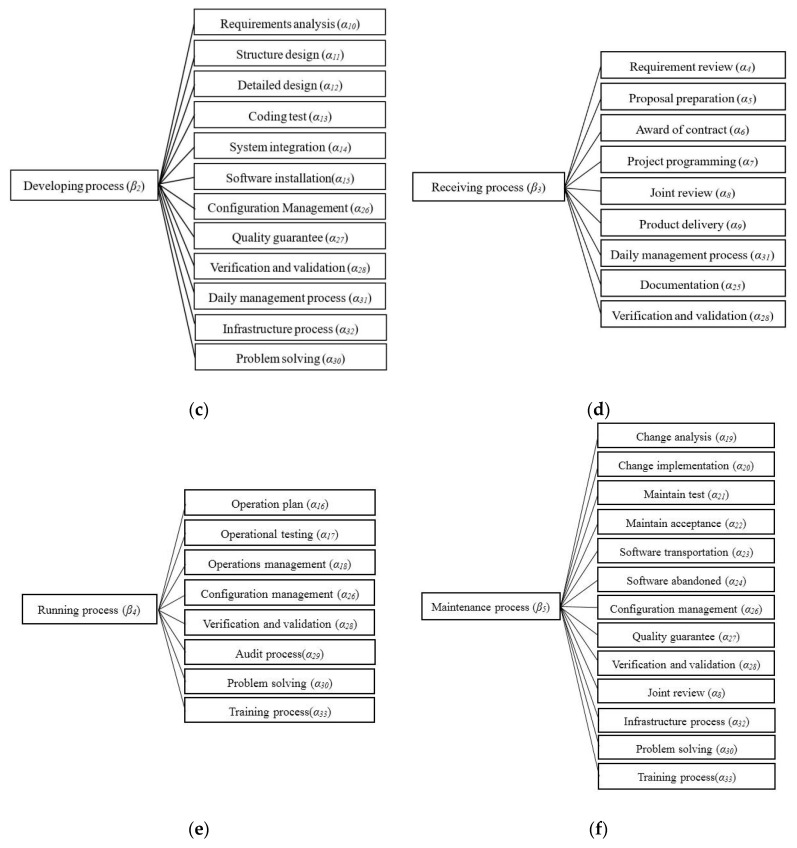
CSCP trustworthiness measurement hierarchical model. (**a**) Main processes; (**b**) Analysis processes; (**c**) Developing processes; (**d**) Receiving processes; (**e**) Running processes; (**f**) Maintenance processes.

SaaS services are characterized by repeatability, rapid scaling, Internet, multi-tenancy, on-demand services, and other features. An increasing number of SaaS services present small size, and are easy to iterate and combine [48]. It is necessary to emphasize more the development process and weaken the supporting and organizing processes, specifically, to consider the latter as a daily behavior and scatter them in each significant department or development process. Therefore, based on ISO/IEC 12207, here the description of specific processes was revised according to the characteristics of cloud services, and the framework of the construction process for cloud services was designed. In CSCP, there was no supporting process or organizing process. Instead, their sub-processes were scattered to the appropriate main process, and the corresponding sub-processes of the main process were modified. In this way, all the sub-processes of the three processes could work together to serve the main process, to facilitate the construction and management of web-delivered services CSCP structure as shown in Figure 1 and Figure 2.

### 3.1. Overall Structure

CSCP is mainly divided into the main process class, supporting process class and organizing process class. The main process is responsible for production, operation, and maintenance of cloud service products. The other two processes serve the main process. The results and data generated during the main process can also be used to improve the initial supporting and organizing processes. The supporting process runs with the main process, providing all kinds of assurance work needed in the main process. Management and staffing of the main process are all constructed and managed by the organizing process. Organizing process is mainly responsible for the construction of infrastructure, personnel composition, and related management rules, as well as the configuration of supporting processes. It starts before the main process and the support process. Figure 1 illustrates the relationship between the three processes.

### 3.2. Main Process, Supporting Process and Organizing Process

The main process is generally used by the participants in developing, running, and maintaining cloud services. A detailed description of each main process is listed in Table 1.

The supporting process provides support for the main process and helps cloud service projects to achieve success and guarantee product quality. It is an effective aid to the sub-processes in the main process category, including documentation process, configuration management process, quality assurance process, verification and validation process, joint review process, audit process, and problem-solving process.

Processes in the organizing process are used to build and implement infrastructure and to make continuous improvement. The infrastructure consists of several related process rules and personnel to improve the coordination between different processes, including management process, infrastructure process, improvement process, and training process.

### 3.3. CSCP Trustworthiness Measurement Hierarchical Model

The quality of cloud service products depends on the overall quality of CSCP. Thus, the activities in the main process have a direct impact on the trustworthiness of the cloud service product. Supporting process and organizing process act directly on the activities in the main process, and hence influence the quality of the final cloud service product. Here, the ISO model was reconstructed, and the sub-activities in supporting process and organizing process were considered as the influencing factors of the sub-activities in the main process. Then the hierarchical model of CSCP trustworthiness measurement was obtained as shown in Figure 2. 

CSCP consists of five main processes: analysis process (*β*_1_), developing process (*β*_2_), receiving process (*β*_3_), running process (*β*_4_) and maintenance process (*β*_5_), as shown in Figure 2a. Their results determine the trustworthiness of CSCP. Supporting process and organizing process, as components or influencing factors of the main process, impact the trustworthiness of each main process. Figure 2b–f provide a hierarchical graph of five main processes respectively and their influencing factors, including a total of 33 risk factors: Defining requirements ($\alpha_{1}$), Bidding preparation ($\alpha_{2}$), Contract preparation ($\alpha_{3}$), Requirement review ($\alpha_{4}$), Proposal preparation ($\alpha_{5}$), Award of contract ($\alpha_{6}$), Project programming ($\alpha_{7}$), Joint review ($\alpha_{8}$), Product delivery ($\alpha_{9}$), Requirements analysis ($\alpha_{10}$), Structure design ($\alpha_{11}$), Detailed design ($\alpha_{12}$), Coding test ($\alpha_{13}$), System integration ($\alpha_{14}$), Software installation ($\alpha_{15}$), Operation plan ($\alpha_{16}$), Operational testing ($\alpha_{17}$), Operations management ($\alpha_{18}$), Change analysis ($\alpha_{19}$), Change implementation ($\alpha_{20}$), Maintain test ($\alpha_{21}$), Maintain acceptance ($\alpha_{22}$), Software transportation ($\alpha_{23}$), Software abandoned ($\alpha_{24}$), Documentation ($\alpha_{25}$), Configuration management ($\alpha_{26}$), Quality guarantee ($\alpha_{27}$), Verification and validation ($\alpha_{28}$), Audit process ($\alpha_{29}$), Problem solving ($\alpha_{30}$), Daily management process ($\alpha_{31}$), Infrastructure process ($\alpha_{32}$), and Training process ($\alpha_{33}$). Section 4 details the calculation of the trustworthiness for each main process and the whole CSCP process according to the hierarchical relationship in Figure 2.

## 4. CSCP Trustworthiness Measurement Method

### 4.1. Related Definitions on CSCP Trustworthiness

**Definition 2** **(Process trustworthiness)** *According to literature [49] and the definition of trustworthiness, trustworthiness of non-functional requirements are mainly manifested in 11 indicators: functional applicability, risk prevention, reliability, security, accuracy, maintainability, performance, ease of use, compatibility, portability and privacy, which are consistent with the indicators of risk concern. Process trustworthiness has a negative correlation with the loss from risk and the uncertainty of risk occurrence. Thus, trustworthiness is calculated by:*

(2) $$T=\;\frac{k_{1}}{U\left(R\right)}+\frac{k_{2}}{L\left(R\right)}$$

*where T is trustworthiness; R is specific risk items; U (R) is the uncertainty of risk occurrence; L(R) is a loss caused by risk; k_1_, k_2_ are constants, representing trustworthiness coefficient, whose values depend on the project or process to be measured. The trustworthiness of the main processes in the hierarchical model is affected by many factors. To calculate the trustworthiness T(γ) and $T\left(\beta_{i}\right)$, it is necessary to obtain the uncertainty functions U(γ) and $U\left(\beta_{i}\right)$ and loss functions L(γ) and $L\left(\beta_{i}\right)$.*

**Definition 3** **(Risk)** *Software process risk includes two essential characteristics: uncertainty and loss impact. Risk can be defined as a triple R = (X, U, L), where X denotes the sub-process or the set of influencing factors that generate risk; U denotes the uncertainty function of risk occurrence; L denotes the risk loss function. The key to risk measurement is to quantify the degree of uncertainty and the degree of loss when there is any risk-related factor [50].*

**Definition 4** **(Risk Uncertainty Function of Main Process)** *The uncertainty of risk occurrence in a process is determined by the risk factors impacting on process trustworthiness. In the CSCP trustworthiness measurement hierarchical model, for the main process β_i_, its risk uncertainty is determined by the threat frequency of each factor $\alpha_{j}$.*

*Let $P\left(\alpha_{j}\right)$ be the threat frequency of the risk factor $\alpha_{j}$, and $P\left(\beta_{i},\alpha_{j}\right)$ be the entropy weight coefficient of $\alpha_{j}$ relative to $\beta_{i}$. Assuming that there are k risk factors in the main process of $\beta_{i}$, the entropy weight coefficient can be calculated by:*

(3) $$P\left(\beta_{i},\alpha_{j}\right)=\frac{1}{\sum_{j=1}^{k}P\left(\alpha_{j}\right)}P\left(\alpha_{j}\right)$$

*Substitute it into the information entropy formula (1), then the following formula is obtained:*

(4) $$U\left(\beta_{i}\right)=-\frac{1}{\log_{2}m}\overset{m}{\underset{j=1}{\sum }}P\left(\beta_{i},\alpha_{j}\right)\log_{2}P\left(\beta_{i},\alpha_{j}\right)$$

$U\left(\beta_{i}\right)\left(0\leq U\left(\beta_{i}\right)\leq 1\right)$ *is the uncertainty function of risk, which denotes the degree of uncertainty of the main process of $\beta_{i}$. Let U(γ) be the uncertainty of CSCP, then:*

(5) $$U\left(\gamma \right)=-\frac{1}{\log_{2}n}\overset{n}{\underset{j=1}{\sum }}P\left(\gamma ,\alpha_{j}\right)\log_{2}P\left(\gamma ,\alpha_{j}\right)$$

**Definition 5** **(Risk loss expectation)** *Risk loss expectation L(x) refers to the degree of loss impact caused by a risk factor. The greater the probability of risk occurrence, the higher the risk it brings to the project; the greater the loss caused by risk, the higher the risk it brings to the project. Therefore, measurement of risk size depends not only on the probability of risk factors but also on its impact on software projects. The Risk loss expectation L(x) can be defined as the product of the occurrence probability P(x) and the degree of loss C(x):*

(6) L(x)=P(x)×C(x)

*In the CSCP trustworthiness measurement hierarchical model, according to formula (5), risk loss $L\left(\alpha_{j}\right)$ caused by a factor is calculated by the product of the probability of occurrence and the degree of loss, that is:*

(7) $$L\left(\alpha_{j}\right)=P\left(\alpha_{j}\right)\times C\left(\alpha_{j}\right)$$

*The formulas for calculating the risk loss of the main process $\beta_{i}$ and the CSCP γ, which are affected by multiple risk factors, as below:*

(8) $$L\left(\beta_{i}\right)=\overset{m}{\underset{j=1}{\sum }}(P\left(\beta_{i},\alpha_{j}\right)\times C\left(\alpha_{j}\right))$$

(9) $$L\left(\gamma \right)=\overset{n}{\underset{j=1}{\sum }}(P\left(\gamma ,\alpha_{j}\right)\times C\left(\alpha_{j}\right))$$

### 4.2. CSCP Trustworthiness Measurement Method

A trustworthiness measurement method for CSCP was proposed based on information entropy. The calculation is detailed as follows:

**Input:** Probability of risk factors *P*(*α_j_*), the degree of loss caused by various factors *C*(*α_j_*).

**Output:** Trustworthiness of the main process *T*(*β_i_*), CSCP trustworthiness *T*(*γ*).

**Step 1:** Establish the evaluation tables as shown in Table 2 and Table 3 below using the Delphi method [51]. Acquire the frequency data *P*(*α_j_*) and loss degree data *C*(*α_j_*) for calculating risk factors according to actual conditions.

**Step 2:** Sort up the raw data acquired. Calculate the frequency and degree of loss of each risk factor in the third tier based on the weight values in Table 2 and Table 3.

**Step 3:** According to the division of the main process in Figure 2, the third level risk factors were classified and normalized. Then, the entropy weight coefficients of risk factors were obtained. Through formula (3), the occurrence frequency *P*(*α_j_*) of the third level risk factors was normalized, and the entropy weight coefficient $P\left(\beta_{i},a_{j}\right)$ relative to the risk of the main process $\beta_{i}$ was obtained. By substituting $P\left(\beta_{i},a_{j}\right)$ and *C*(*α_j_*) into formulas (4), (7) and (8) respectively, the uncertainty degree $U\left(\beta_{i}\right)$ and loss degree $L\left(\alpha_{j}\right)$ and $L\left(\beta_{i}\right)$ of the risk of the first main process were calculated. 

**Step 4:** From formula (5), the occurrence frequency *P*(*α_j_*) of all the third level risk factors was normalized, and the entropy weight coefficient P(γ) of the whole process relative to CSCP was obtained. By substituting P(γ) and *C*(*α_j_*) into formulas (5) and (9) respectively, the uncertainty degree U(γ) and loss degree L(γ) were calculated. 

**Step 5:** The uncertainty degree $U\left(\beta_{i}\right)$ and loss degree $L\left(\beta_{i}\right)$ of the risk of each main process were substituted into formula (2) to obtain the trustworthiness of each main process $T\left(\beta_{i}\right)$. The trustworthiness T(γ) of CSCP was acquired by substituting uncertainty degree U(γ) and loss degree L(γ) of CSCP risk into formula (2). 

## 5. Case Study and Analysis

### 5.1. Case Study

Y is a small and medium-sized software company mainly engaged in mobile applications (app) and SaaS services in a cloud computing environment. In different product development processes, it is subject to occasional failures or low-quality products, which may be attributed to various reasons. In the case study, the trustworthiness of the company’s service development process was calculated, providing a reference for its follow-up process improvement work. Due to the privacy of the service development documents, a questionnaire was designed containing 33 risk factors according to the model in Figure 2. The frequency and extent of risk occurrence and loss included in the questionnaire were scored according to Table 2 and Table 3. A total of 15 employees from the company participated in the survey, covering a variety of roles including systems analysts, designers, developers, testers, maintainers, managers, and trainers. These participants were graded anonymously according to the analysis report of failed products and actual work experience, and the scores were used as the original data for later calculation.

The specific calculation steps are as follows:

**Step 1:** Collect the questionnaires and count the scoring results. The results are shown in Table 4.

**Step 2:** Calculate the risk frequency $P\left(\alpha_{j}\right)$ and loss degree weight *C*(*α_j_*) of the risk factors according to the following formulas, and the results are shown in Table 5:

(10) $$\left(a_{j}\right)=\left(\sum_{i=1}^{5}(\omega_{i}\times k_{i}\right))/n$$

where $\omega_{i}$ is the weight value of an influencing factor in Table 2 and Table 3; $k_{i}$ is the number of people choosing the weight value of the influencing factor, and *n* is the total number of people who participated in the questionnaire survey. Here the value of n is 15.

**Step 3:** According to Figure 2, calculate the entropy weight coefficient $P\left(\beta_{i},a_{j}\right)$ of each main process. Then substitute each $P\left(\beta_{i},a_{j}\right)$ into Equations (4), (7) and (8) respectively and calculate the risk uncertainty $U\left(\beta_{i}\right)$ and loss degree $L\left(\beta_{i}\right)$ of each main process. The results are shown in Table 6. 

**Step 4:** Using Equation (5), normalize the occurrence frequency *P*(*α_j_*) of the risk factors and obtain the entropy weight coefficient P(γ) of CSCP. Then substitute P(γ) and *C*(*α_j_*) into Equations (5) and (9) respectively, and calculate the risk uncertainty U(γ) and loss degree L(γ). The results are shown in Table 6.

**Step 5:** To simplify the calculation, after receiving the consent of Company Y, set the trustworthiness coefficients *k*_1_ and *k*_2_ as 1. Then, substitute the uncertainty degree $U\left(\beta_{i}\right)$ and loss degree $L\left(\beta_{i}\right)$ of the risk of each main process into Equation (2). Obtain the trustworthiness of each main process $T\left(\beta_{i}\right)$. Substituting the uncertainty degree U(γ) and loss degree L(γ) of CSCP into Equation (2), the trustworthiness of CSCP T(γ) is obtained. The results are shown in Table 7.

### 5.2. Analysis

**Figure 3:**
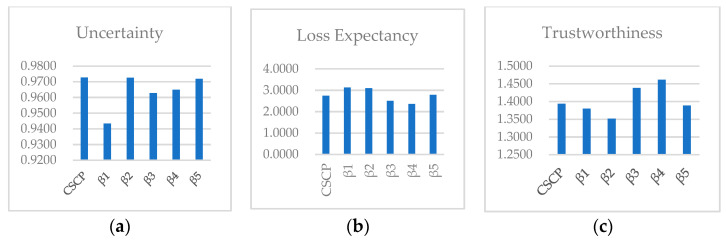
Results. (**a**) Uncertainty of CSCP; (**b**) Loss Expectancy of CSCP; (**c**) Trustworthiness of CSCP.

From Table 6 and Table 7, the final calculation results were summed up, as shown in Figure 3. The proposed method was mainly designed to measure the trustworthiness of the software construction process within relevant organizations. It was hoped to help organizations identify weaknesses within the organization, thereby improving and optimizing the construction process. The construction process of Y Company was analyzed mainly from three dimensions: trustworthiness, uncertainty and risk expectation.

(1) Process trustworthiness analysis. It is found in Figure 3 that $T\left(\beta_{2}\right)<T\left(\beta_{1}\right)<T\left(\beta_{5}\right)<T\left(\gamma \right)<T\left(\beta_{3}\right)<T\left(\beta_{4}\right)$. In running process and receiving process, the user acquires and uses the service. The trustworthiness of these two processes was observed to be higher than that of the whole process T(γ), showing better quality, stability and safety than those of the other three processes. Hence, the two processes were not responsible for the failure of the projects. Also, the maintenance process was not the cause of project failure either, owing to its close trustworthiness to that of T(γ). All the above three processes performed after product development, delivery and use. Therefore, it was concluded that the processes of user acceptance, product operation, and maintenance were not the causes of project failure, but the development process and analysis process with the lowest trustworthiness. In the future, Y Company should carefully analyze the existing problems in development and analysis processes, strengthen process management and improve the business level of personnel in these two processes. The proposed method can be used to conduct a more in-depth analysis of the specific problems. 

(2) Process uncertainty analysis. The results showed $U\left(\beta_{1}\right)<U\left(\beta_{3}\right)<U\left(\beta_{4}\right)<U\left(\beta_{5}\right)<U\left(\beta_{2}\right)<U\left(\gamma \right)$. Uncertainty of process risk refers to the probability distribution of the risk factors occurrence. Uncertainty reflects the difficulty of risk control. The higher the uncertainty is, the less obvious the cause of risk is and the more difficult the maintenance and control of risk will be. Y Company exhibited low uncertainty of analysis process and receiving process, indicating that the risk factors of these two processes were relatively obvious and well controlled. In other words, under the risk of these two processes, the cause could be found more clearly and directly. By contrast, the risk factors in maintenance process and development process remained unclear, the distribution of which was relatively average. Hence it was difficult to quickly identify the causes of problems in these two processes.

(3) Loss expectation analysis. It was observed that $L\left(\beta_{4}\right)<L\left(\beta_{3}\right)<L\left(\gamma \right)<L\left(\beta_{5}\right)<L\left(\beta_{2}\right)<L\left(\beta_{1}\right)$. In other words, risks in running process and receiving process tended to cause the lowest loss. It can be explained that the main participants in these two processes are users. Most of the problems are caused by poor user operation and management. Hence Y Company was subject to a small impact of project failure. Yet, the problems in the development process and analysis process were more likely to cause great losses. In particular, a problem in the analysis process may lead to the failure of the whole project. Thus, more attention should be paid to the analysis process.

The company hired professional evaluation agencies to test its internal management, research and development levels, with different evaluation dimensions and methods. The results were consistent and found that there were major defects in the company’s demand analysis, definition process and development process, which is in line with the results of this paper. In addition, during the questionnaire survey, communication with the company’s internal staff and related leaders found that the company did not pay enough attention to the research on the preliminary demand. Also, its development team was very young and changed its members frequently. On the other hand, it maintained relatively standardized regulation of sales-related and other management, which is consistent with the results of this paper.. In terms of methodology, information entropy theory serves as a measurement tool. The research on measuring product quality and risk has achieved positive results. Therefore, it is feasible and theoretically and practically important to detect weaknesses and improve the construction process by applying information entropy theory and method to process measurement.

Based on the frequency of problems occurring in the sub-procedure of CSCP and the consequent loss degree, this paper tries to calculate the trustworthiness of the cloud service establishment process. Any change of *P(αj)* and *C(αj)* in the sub-procedure will give rise to the change of credibility including its parent process, which will be reflected in the final credibility results. Thus, it is convenient for users to discover links with problems and measure the improvement effects. However, this paper mainly calculates the relative credibility of the enterprise’s internal process, which is suitable for those that have regional problems but have no idea where they are, and the case company is a typical example of them, and such enterprises account for the vast majority of SMEs. But for enterprises whose integral level is relatively balanced or those to be specified in their process level, if our method is chosen, other data need to be introduced so as to establish the standard for measurement.

### 5.3. Comparisons

#### 5.3.1. Comparison with AHP

Analytic Hierarchy Process (AHP) is an evaluation- and decision-making method that combines qualitative and quantitative analyses, which can be applied to multi-objective, multi-element, multi-level problem solving. CSCP trustworthiness measure just meets such requirements. AHP can be used to measure a specific CSCP. However, AHP first requires an entire evaluation system to be established. In order to find out the cause of a problem, it is necessary to comprehensively find out the factors that affect the problem. In addition, the AHP method involves multi-person scoring and calculation, with corresponding consistency detection operations, but the measurement results are still highly subjective, as a result of varied environments and statuses of the participants. Moreover, if multiple processes or entirety are to be measured, each sub-process should be analyzed separately to find the influencing factors. Also, the relationship between sub-processes should be considered. As the entire development process contains many sub-processes, comprehensive measurement requires a large amount of work, and failure to offer complete or correct measurement results in compromised trustworthiness. In addition, AHP can be applied to other processes. However, relevant measurement models are poorly compatible to construction process, management process, and support process, and other processes of different organizations. Therefore, it is necessary to re-analyze and re-select the influencing factors. Further, as participants are varied and very subject, the measurement results may be highly unstable.

In this paper, the CSCP framework is based on ISO/IEC 12207. Analysis and summary of many cloud service construction processes found that the management process and support process of ISO/IEC 12207 are split and refined and scattered into each main process, which basically covers the entire process of existing cloud service development. In terms of specific application, the required processes and sub-processes can be selected and measurement can be conducted within the CSCP framework. Or the selection might be skipped to directly measure all the processes involved in the entire CSCP framework (irrelevant processes will be scored zero), which is highly compatible and plastic. Furthermore, the method proposed in this paper is easy to input, which only requires two parameters (risk occurrence frequency and loss degree). The input quantity is small and easy to obtain. Moreover, in this paper, risk occurrence frequency and loss degree data can only be acquired by questionnaires, for the data is confidential. In reality, if the relevant data of previous projects are offered, the two input parameters required in the method can be obtained by data mining, etc. Therefore, it is ensured that the method is objective.

#### 5.3.2. Comparison with CMM/CMMI

CMM/CMMI is a set of modes and methods for the management, improvement and evaluation of software processes. It stipulates the characteristics of various levels of software development process capabilities and the goals for improvement. It can help software companies manage and improve software engineering processes, and enhance development and improvement capabilities, thereby developing high quality software timely within budget. CMM/CMMI evaluation requires the following: a dedicated evaluation team; an established CMM/CMMI evaluation system model to classify the development process into key processes and identify the main activities for later; maturity questionnaires designed based on processes and activities; on-site visits; generated survey lists; evaluation and conclusions. Therefore, CMM/CMMI is a complex engineering system that requires comprehensive cooperation by all parts of the company, a large amount of capital, manpower, material resources and time. It is not for small and medium-sized enterprises and enterprises with poor management.

The small size of cloud services makes its development and management process flexible, but CMM/CMMI is a large-scale measurement system that does not suit small-scale software services, for it requires heavy investment, high cost and is highly complex, with poor adaptability to small-scale processes. CMM/CMMI is a comprehensive and complete method for judging whether processes like organization, development, management, meet relevant benchmark requirements. Small and medium-sized enterprises (SMEs), especially cloud service development organizations, are more concerned about the weaknesses and risks in the development and management processes of the method. As factors like personnel and environment change, its development and management processes should be frequently evaluated. Therefore, SMEs need a measurement method that is simple, easy, low-cost, low-investment and can be frequently conducted. With the development of enterprises, the CMM/CMMI method may be introduced according to the actual needs when enterprises grow to a certain extent. The method can measure and evaluate the overall processes, thereby comprehensively improving processes. Daily management and development processes of enterprises need a small-scale method for self-test and self-evaluation, at which the research in this paper is aimed.

#### 5.3.3. Summary

All three methods can measure the quality of cloud service construction process. However, the three methods focus on different areas, with significant differences in usability, objectivity, versatility, functionality and cost. To facilitate the description, the method in this paper is abbreviated to information entropy method, IEM.

In the aspect of usability, (*U*(*x*)), it is mainly reflected in Data Acquisition Difficulty (DAD), Operational Process Complexity (OPC) and Readability of Results (RR). According to what is described in Section 5.3.1 and Section 5.3.2, Table 8 is formed. In brief, the usability degree of a certain main method has an inverse correlation with DAD and OPC. Thus, we can get the comparison result of the three methods in usability: *U*(IEM) > *U*(AHP) > *U* (CMM/CMMI).

In the aspect of objectivity (*O*(*x*)), it is mainly determined by the objectivity of the data for measurement, the model and the operational process. In terms of IEM method, the input data include the frequency of risks in the previous projects and the loss degree caused by risks in different processes, all of which can be summarized from the previous data. At the same time, the model is mainly based on international standards and development practice, whose operational process is actually the data computational process, so it is of the higher objectivity. CMM/CMMI is a method measured by others, which possesses both a mature measurement system and the acquisition method of necessary data. But the data acquisition process and the operational process need the coordination of the personnel in the whole process, so its objectivity is inferior to IEM method. In terms of AHP method, the input data completely come from grading of the personnel. In spite of its consistency detection mechanism, its objectivity is still greatly affected. Besides, its process measurement model should be established temporarily by the measurement personnel, whose reference frame and establishment process are easy to be affected by human factors. Thus, its objectivity is lower than CMM/CMMI. The comparison result of the three methods in objectivity is as follows: *O*(IEM) > *O*(CMM/CMMI) > *O*(AHP).

In the aspect of versatility (*V*(*x*)) and functionality (*F*(*x*)), CMM/CMMI is a mature international measurement standard, which can not only help find out the weak links and analyze causes, but also offer the grades of an organization’s ability maturity, so it is suitable for varieties of software development institutions or organizations. As to the IEM method, it is a lightweight method aimed at small and medium-sized enterprises, which can discover the weak links in development and management, but is powerless in determining the ability maturity grades. AHP is a universal method, but in the field of process measurement, aimed at different institutions, it is necessary to first establish the process model. However, there are huge differences in the development and management process of different institutions. Thus, the measurement model of one institution is unlikely to apply to other institutions, whose versatility is not quite good in the process measurement field. AHP finally offers subordinate grades for process credibility, but for small and medium-sized enterprises, in order to better reduce risks and guarantee quality, what they pay close attention to is not which grade their management level is, but where their weak link is. The comparison results of the three methods in versatility and functionality is as follows: *V*(CMM/CMMI) > *V*(IEM) > *V*(AHP), *F*(CMM/CMMI) > *F*(IEM) > *F*(AHP).

In the aspect of cost (*Cost*(*x*)), for CMM/CMMI, professional measurement teams are required to participate in the whole process of development and management, which involves a huge amount of capital, time and energy. AHP method needs to employ experts to engage in the establishment, grading and calculation of the measurement model, which also requires of certain investment. As to the IEM method, the input is based on previous data or experience, which can realize measurement through statistical analysis of the frequencies and loss degree of previous risks. In addition, with the usage of the mining method in the later period, it will further reduce the cost and time in data statistics. Thus, it is a method with the lowest cost. The comparison result of the three methods in cost is as follows: *Cost*(CMM/CMMI) > *Cost*(AHP) > *Cost*(IEM).

Based on the comparisons above, we get the specific results which are shown in Table 9, Table 10 and Table 11.

SMEs are flexible in software development within the cloud computing environment. However, they lack a set of effective methods for measuring process management. Traditional self-evaluation through project summary meetings fails to find the real process problems in most cases. CMM/CMMI, AHP and other methods may deliver good and comprehensive performance, but they are difficult to operate, and most of them require additional cost. Therefore, they cannot be frequently used by SMEs. The method in this paper is aimed at finding a simple, easy-to-use, low-cost process self-test method for specific cloud services and even software service development organizations. It finds out weaknesses through self-test and self-evaluation on a regular basis, thereby continuously improving development and management processes. It has significant advantages in terms of operation and cost for organizations with variable organizational structures.

## 6. Conclusions

With the advent and popularization of cloud computing, cloud services have gradually become the main computing mode. With increasingly more cloud services in the market, their trustworthiness becomes the key to product selection and largely depends on the process of constructing cloud services. Hence, the trustworthiness measurement of CSCP becomes crucial for the trustworthiness of cloud services. It can help cloud service developers identify the weaknesses in the development process and the main risk factors causing losses. Eventually, it benefits the organizations by improving and optimizing the development process of cloud services, which is conducive to enhancing the quality of cloud services.

CMM and CMMI methods are costly, long-term and extremely complicated for small and medium-sized organizations. This paper presents an objective, quantitative, simple and effective method for CSCP trustworthiness measurement. Firstly, combined with the concepts and characteristics of ISO/IEC 12207 and CSCP, the main process of cloud service construction and its influencing factors were obtained, and a hierarchical model of trustworthiness measurement for CSCP was established. Then the trustworthiness of CSCP was defined, and the uncertainty and risk loss expectation of CSCP was calculated. Based on the uncertainty and risk loss expectation, the trustworthiness of CSCP was calculated. Finally, through a case study, the trustworthiness of the company’s development process was calculated, which verified the feasibility and correctness of the proposed method.

A simple and feasible method was proposed here to measure the trustworthiness of CSCP. Yet, it is only helpful for process measurement and improvement within a specific organization. Due to data sources and influencing factors, horizontal comparisons between different agencies are needed in further research. Although the participants undertake a variety of roles in the company, objectivity of the data used in the calculation is still impacted to a certain extent. It can be improved by obtaining the frequency of the risk factors and the loss degree caused by the factors through past analysis reports or operational data of the system. 

## Figures and Tables

**Table 1:** Main Process.

Processes	Descriptions
Analysis process (*β*_1_)	Buyers’ activities to acquire systems, software products or software services, including definition, analysis of requirements, tender preparation, contract preparation, acceptance, and acceptance, etc.
Developing process (*β*_2_)	Developers define and develop activities for software products, including system requirements analysis, structural design, detailed design, coding and testing, system integration, software installation, acceptance, etc.
Receiving process (*β*_3_)	Activities of suppliers and demanders in supplying systems, software or service products, including reviewing requirements, preparing bids, signing contracts, formulating and implementing project plans, conducting reviews and evaluations, delivering products, etc.
Running process (*β*_4_)	Operators’ activities of providing computer system services to their users in specified environments include formulating and implementing operation plans, running tests, system operation, providing help and consultation to users, etc.
Maintenance process (*β*_5_)	Maintainers provide activities to maintain software and service products, including analysis of problems and changes, implementation of changes, maintenance review, maintenance acceptance, software migration, software exit, etc.

**Table 2:** The assignment table of risk frequency *P*(*α_j_*).

Weight	Level	Description
5	Very high	The frequency of risk caused by this factor is very high, and it is inevitable in practice.
4	High	The frequency of risk caused by this factor is high, and it will happen in most cases.
3	Medium	The frequency of risk caused by this factor is general and may occur in some cases.
2	Low	The frequency of risk caused by this factor is low, and it will occur in a few cases.
1	Very low	The frequency of risk caused by this factor is very low, and it hardly happens in practice.

**Table 3:** The assignment table of risk loss *C*(*α_j_*).

Weight	Level	Description
5	Very high	Once this risk occurs, it will cause devastating losses.
4	High	The impact of this risk is more significant and the maintenance fund is higher.
3	Medium	The economic losses and impacts caused by this risk are general.
2	Low	The impact of this risk is small and the maintenance fund is low.
1	Very low	The impact of this risk is negligible and requires little maintenance.

**Table 4:** Statistical results of *P*(*α_j_*) and *C*(*α_j_*).

*α_j_*	*P*(*α_j_*)					*C*(*α_j_*)					*α_j_*	*P*(*α_j_*)					*C*(*α_j_*)				
	1	2	3	4	5	1	2	3	4	5		1	2	3	4	5	1	2	3	4	5
$\alpha_{1}$	**0**	0	1	12	2	0	0	7	7	1	$\alpha_{18}$	1	2	8	4	0	1	1	6	6	1
$\alpha_{2}$	10	3	2	0	0	9	6	0	0	0	$\alpha_{19}$	6	8	1	0	0	1	1	5	7	1
$\alpha_{3}$	11	2	2	0	0	1	0	1	10	3	$\alpha_{20}$	2	2	2	2	7	3	5	4	2	1
$\alpha_{4}$	0	6	8	1	0	2	11	2	0	0	$\alpha_{21}$	10	3	1	1	0	8	5	0	0	2
$\alpha_{5}$	9	6	0	0	0	6	2	0	2	5	$\alpha_{22}$	14	1	0	0	0	13	1	1	0	0
$\alpha_{6}$	12	3	0	0	0	11	3	0	0	1	$\alpha_{23}$	2	11	2	0	0	0	1	9	5	0
$\alpha_{7}$	0	1	5	8	1	0	1	1	10	3	$\alpha_{24}$	13	2	0	0	0	1	0	11	2	1
$\alpha_{8}$	6	7	2	0	0	11	4	0	0	0	$\alpha_{25}$	13	1	1	0	0	1	1	6	6	1
$\alpha_{9}$	13	2	0	0	0	11	4	0	0	0	$\alpha_{26}$	3	4	7	1	0	6	1	1	6	1
$\alpha_{10}$	1	1	7	5	1	0	0	4	8	3	$\alpha_{27}$	9	5	1	0	0	0	0	1	7	7
$\alpha_{11}$	9	3	3	0	0	0	0	2	3	10	$\alpha_{28}$	9	5	1	0	0	1	2	10	2	0
$\alpha_{12}$	14	1	0	0	0	3	6	5	1	0	$\alpha_{29}$	2	2	7	2	2	10	5	0	0	0
$\alpha_{13}$	3	4	7	1	0	9	5	1	0	0	$\alpha_{30}$	2	5	6	1	1	1	1	7	5	1
$\alpha_{14}$	13	2	0	0	0	11	3	0	0	1	$\alpha_{31}$	6	7	1	1	0	4	10	1	0	0
$\alpha_{15}$	13	2	0	0	0	1	1	0	10	3	$\alpha_{32}$	13	1	1	0	0	0	0	1	1	13
$\alpha_{16}$	13	2	0	0	0	10	4	1	0	0	$\alpha_{33}$	13	2	0	0	0	11	3	1	0	0
$\alpha_{17}$	10	4	1	0	0	11	4	0	0	0											

**Table 5:** $P\left(a_{j}\right)$ and *C*(*α_j_*).

$\alpha_{j}$	*P*(*α_j_*)	*C*(*α_j_*)	$\alpha_{j}$	*P*(*α_j_*)	*C*(*α_j_*)
$\alpha_{1}$	4.0667	3.6000	$\alpha_{18}$	3.0000	3.3333
$\alpha_{2}$	1.4667	1.4000	$\alpha_{19}$	1.6667	3.4000
$\alpha_{3}$	1.4000	3.9333	$\alpha_{20}$	3.6667	2.5333
$\alpha_{4}$	2.6667	2.0000	$\alpha_{21}$	1.5333	1.8667
$\alpha_{5}$	1.4000	2.8667	$\alpha_{22}$	1.0667	1.2000
$\alpha_{6}$	1.2000	1.4667	$\alpha_{23}$	2.0000	3.2667
$\alpha_{7}$	3.6000	4.0000	$\alpha_{24}$	1.1333	3.1333
$\alpha_{8}$	1.7333	1.2667	$\alpha_{25}$	1.2000	3.3333
$\alpha_{9}$	1.1333	1.2667	$\alpha_{26}$	2.4000	2.6667
$\alpha_{10}$	3.2667	3.9333	$\alpha_{27}$	1.4667	4.4000
$\alpha_{11}$	1.6000	4.5333	$\alpha_{28}$	1.4667	2.8667
$\alpha_{12}$	1.0667	2.2667	$\alpha_{29}$	3.0000	1.3333
$\alpha_{13}$	2.4000	1.4667	$\alpha_{30}$	2.6000	3.2667
$\alpha_{14}$	1.1333	1.4667	$\alpha_{31}$	1.8000	1.8000
$\alpha_{15}$	1.1333	3.8667	$\alpha_{32}$	1.2000	4.8000
$\alpha_{16}$	1.1333	1.4000	$\alpha_{33}$	1.1333	1.3333
$\alpha_{17}$	1.4000	1.2667			

**Table 6:** $U\left(\beta_{i}\right)$, $L\left(\beta_{i}\right)$ and U(γ), L(γ).

Processes	Uncertainty (*U*)	Loss Expectancy (*L*)
Analysis process $\beta_{1}$	0.9434	3.1273
Developing process $\beta_{2}$	0.9726	3.0945
Receiving process $\beta_{3}$	0.9627	2.5051
Running process $\beta_{4}$	0.9649	2.3534
Maintenance process $\beta_{5}$	0.9719	2.7832
CSCP γ	0.9727	2.7365

**Table 7:** $T\left(\beta_{i}\right)$ and T(γ).

Processes	Trustworthiness (*T*)
Analysis process $\beta_{1}$	1.3798
Developing process $\beta_{2}$	1.3514
Receiving process $\beta_{3}$	1.4380
Running process $\beta_{4}$	1.4613
Maintenance process $\beta_{5}$	1.3882
CSCP γ	1.3935

**Table 8:** Comparison in Usability.

	Comparison Results
**DAD**	IEM < AHP < CMM/CMMI
**OPC**	IEM = AHP < CMM/CMMI
**RR**	IEM >= AHP > CMM/CMMI

**Table 9:** Comparison with AHP and CMM/CMMI (1).

	Comparison Results
**Usability**	IEM > AHP > CMM/CMMI
**Objectivity**	IEM > CMM/CMMI > AHP
**Versatility**	CMM/CMMI > IEM > AHP
**Functionality**	CMM/CMMI > IEM > AHP
**Cost**	CMM/CMMI > AHP > IEM

**Table 10:** Comparison with AHP and CMM/CMMI (2).

	Application Scenario
**IEM**	To find relative weaknesses for continuous improvement by self-test
**AHP**	To help with decision making by self-test
**CMM/CMMI**	To set benchmark and to judge whether it is up to standard by other tests

**Table 11:** Comparison with AHP and CMM/CMMI (3).

	Usability	Objectivity	Versatility	Functionality	Cost
**IEM**	High	High	Modest	Modest	Low
**AHP**	Modest	Low	Low	Low	Modest
**CMM/CMMI**	Low	Modest	High	High	High
